# *ESKIMO1* Disruption in Arabidopsis Alters Vascular Tissue and Impairs Water Transport

**DOI:** 10.1371/journal.pone.0016645

**Published:** 2011-02-01

**Authors:** Valérie Lefebvre, Marie-Noëlle Fortabat, Aloïse Ducamp, Helen M. North, Alessandra Maia-Grondard, Jacques Trouverie, Yann Boursiac, Gregory Mouille, Mylène Durand-Tardif

**Affiliations:** 1 Institut Jean-Pierre Bourgin UMR1318 INRA-AgroParisTech, INRA Centre de Versailles-Grignon, Versailles, France; 2 Ecophysiologie Végétale, Agronomie et nutritions N, C, S, UMR INRA-UCBN, Université de Caen Basse-Normandie, Caen, France; 3 Biochimie et Physiologie Moléculaire des Plantes, Institut de Biologie Intégrative des Plantes, UMR 5004 CNRS/UMR 0386 INRA/Montpellier SupAgro/Université Montpellier 2, Montpellier, France; University of California, Davis, United States of Ameica

## Abstract

Water economy in agricultural practices is an issue that is being addressed through studies aimed at understanding both plant water-use efficiency (WUE), i.e. biomass produced per water consumed, and responses to water shortage. In the model species *Arabidopsis thaliana*, the *ESKIMO1* (*ESK1*) gene has been described as involved in freezing, cold and salt tolerance as well as in water economy: *esk1* mutants have very low evapo-transpiration rates and high water-use efficiency. In order to establish ESK1 function, detailed characterization of *esk1* mutants has been carried out. The stress hormone ABA (abscisic acid) was present at high levels in *esk1* compared to wild type, nevertheless, the weak water loss of *esk1* was independent of stomata closure through ABA biosynthesis, as combining mutant in this pathway with *esk1* led to additive phenotypes. Measurement of root hydraulic conductivity suggests that the *esk1* vegetative apparatus suffers water deficit due to a defect in water transport. *ESK1* promoter-driven reporter gene expression was observed in xylem and fibers, the vascular tissue responsible for the transport of water and mineral nutrients from the soil to the shoots, via the roots. Moreover, in cross sections of hypocotyls, roots and stems, *esk1* xylem vessels were collapsed. Finally, using Fourier-Transform Infrared (FTIR) spectroscopy, severe chemical modifications of xylem cell wall composition were highlighted in the *esk1* mutants. Taken together our findings show that ESK1 is necessary for the production of functional xylem vessels, through its implication in the laying down of secondary cell wall components.

## Introduction

The environmental conditions in which plants live vary constantly, during the day, with the seasons and as a consequence of climate. Plants are sessile organisms that are constrained to complete their live-cycle in one place and therefore must protect themselves against adverse conditions: their growth depends on their responses to the environment.

Plants perceive variations in the environment and react, in accordance to the nature and the intensity of the stress (reviewed in [1]). In response to abiotic stress, many genes are either down-regulated, mostly genes involved in growth, or activated at the transcriptional level [2], [3], [4], [5], [6]. Up-regulated genes can propagate the stress signal (transcription factors, hormone synthesis…), be involved in homeostasis (osmoticum synthesis, ions and water transport, molecule protection by chaperones…) or damage limitation (detoxification, antioxidants…) (reviewed in [7], [8]). As a result, there is a constant readjustment of the balance between growth arrest in order to protect and save the organism and growth maintenance at the risk of exhausting water or nutrient supplies (e.g. [9]). The regulation of stomata aperture according to water availability is a good illustration of this equilibrium. Stomata are the pores through which plants lose water through evapo-transpiration and acquire CO_2_ for carbon fixation by photosynthesis: closing stomata economizes water, but reduces growth as CO_2_ influx is limited. Water depletion induces stomata closure, through an increase of the stress hormone ABA [10], [11].

The *ESK1* gene was initially identified by map-based cloning of the *Arabidopsis thaliana esk1-1* mutation obtained using an *in vitro* screen for constitutive freezing tolerance [12]. Subsequently, it was shown that when *esk1* plants were grown in soil, they were more tolerant to freezing than wild type after a period of acclimation [13], [14]. *esk1* mutant phenotypes are those of a stressed plant: reduced stature and relative water content (RWC) compared to wild type [12], [14], [15], it accumulates high levels of stress metabolites that are thought to maintain the internal osmotic status, such as the osmoticum proline [12], [15] and its transcriptome resembles that of drought-stressed wild type plants [14]. Furthermore, the evapo-transpiration of *esk1* mutants is severely reduced and their water use efficiency (WUE) i.e. the amount of carbon incorporated in biomass per water used, is higher than wild type.

The stressed phenotypes of *esk1* led us to examine whether *ESK1* was implicated in the action of the stress hormone ABA. We have examined ABA production in *esk1* mutants and the phenotypes of double mutants affected in ABA responses and *esk1*. Then, we addressed the origin of the very low evapo-transpiration of *esk1*. We observed that cross sections of *esk1* vascular tissue had an “*irregular xylem*” (*irx*) phenotype. Analysis of the xylem cell wall in *esk1* mutants highlighted significant modifications in chemical composition compared to the wild type. Furthermore, we assessed *esk1* root-hydraulic conductivity. Taken together, our results indicate that ESK1 is necessary for the formation of the functional xylem required for water transport to aerial plant tissues.

## Results

### ABA levels are altered in *esk1* plants

**Figure 1:**
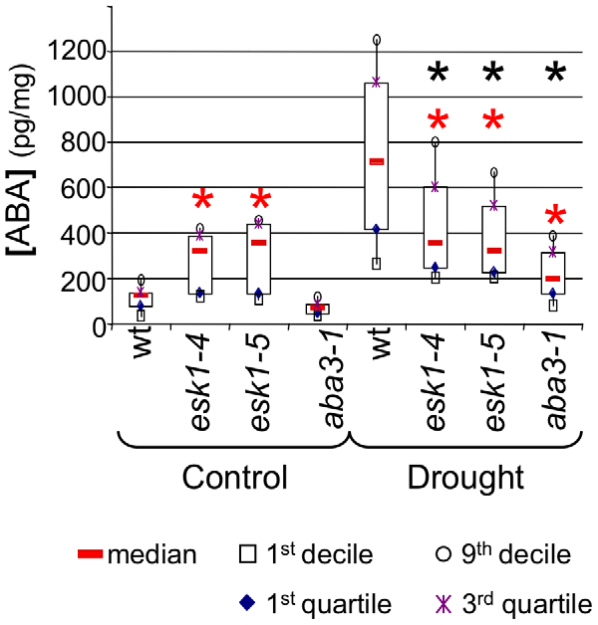
Basal ABA levels are high in *esk1* mutants. Box-plot of [ABA] determined for wild type Col-0 (wt), *aba3-1*, *esk1-4* and *esk1-5* under control and mild drought conditions. The [ABA] is reported as pg/mg of dry weight. Each box-plot was produced from results for 11 or 12 measurements. Red * indicates significant difference with wild type for the same treatment and black * indicates significant genotype x treatment interaction (*p*-value<0.05) for each pair of wild type and the other three genotypes.

*esk1* mutants were previously described as freezing [12], cold and salt tolerant [14], [15]. A common factor in these stress responses is the accumulation of the phytohormone ABA [16]. In order to determine whether ABA was implicated in inducing the stressed *esk1* state, ABA contents were measured in rosettes of wild type and two *esk1* mutants (*esk1-4* and *esk1-5*) submitted or not to mild drought (Figure 1) and compared to the ABA-deficient mutant *aba3-1*, affected in the sulfuration of the molybdenum cofactor required for the last steps of ABA biosynthesis [17], [18]. ABA measurements from the two *esk1* mutants were similar as expected for null mutant, with or without drought treatment (Table S1, LSD tests).

In control conditions, ABA contents were significantly higher in *esk1* mutants than either wild type or *aba3-1*, the latter being due to its defect in ABA biosynthesis [17]. Increased in ABA production is associated with stress and is in accord with *esk1* mutants being stressed *per se.* As previously described, ABA contents were significantly higher in drought conditions for wild type and *aba3-1*, however, ABA levels in *esk1* mutants were the same as those in non-stressed conditions (Table S1, 1D ANOVA). The slight increase in ABA levels in *aba3-1* plants in these conditions is probably because this is not a null allele [19].

We found significant genotype x treatment interactions for the wild type and the other three genotypes (Figure 1, black stars and Table S1, 2D ANOVA), but no interactions were significant when analyzing *esk1* and *aba3-1* (Table S1, 2D ANOVA). These results demonstrate that *esk1* mutants respond differently to drought stress than wild type.

### ESK1, ABA3 and likely OST1 act in independent pathways

**Figure 2:**
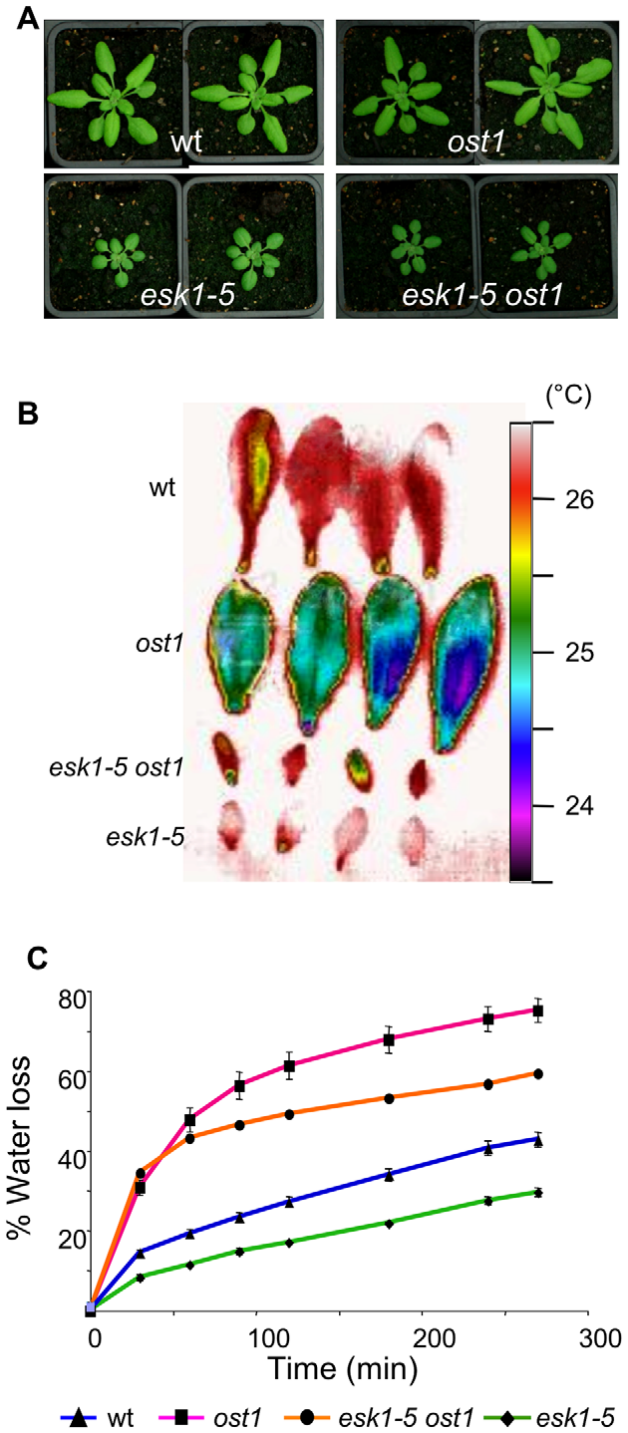
*esk1-5 ost1* double mutant phenotypes. A. Two representative plants of each genotype grown in standard non-stressed conditions: wild type (wt), *ost1*, *esk1-5* and *esk1-5 ost1* double mutant. B. False color infrared image of detached leaves from 4 week-old plants, 2 leaves from 2 plants were observed for each genotype. C. Rapid dehydration of detached rosettes from 4 week-old plants. Water loss was determined as % of initial fresh weight. Error bars represent standard error values (n = 4).

**Figure 3:**
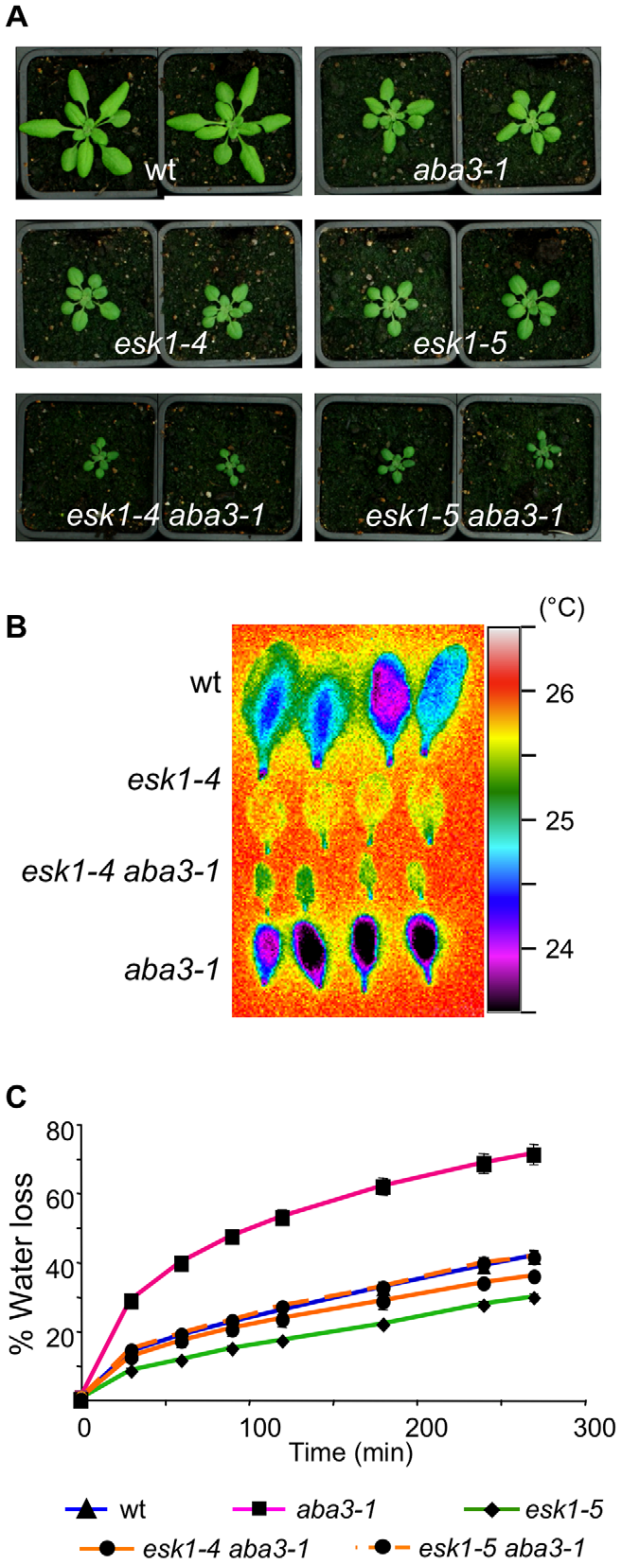
*esk1 aba3-1* double mutants phenotypes. A. Two representative plants of each genotype grown in standard non-stressed conditions: wild type (wt), *aba3-1*, *esk1-4*, *esk1-5*, *esk1-4 aba3-1* and *esk1-5 aba3-1* double mutants. B. False color infrared image of detached leaves from 4 week-old plants, 2 leaves from 2 plants were observed for each genotype. C. Rapid dehydration of detached rosettes from 4 week-old plants. Water loss was determined as % of initial fresh weight. Error bars represent standard error values (n = 4).

To determine whether the higher ABA levels observed in *esk1* mutants were a direct or indirect consequence of ESK1 function, double mutants were generated with either *aba3-1* or the ABA signaling mutant *ost1/snrk2e.* OST1 is a SnRK2 protein kinase that acts in an early step of the ABA signaling pathway in guard cells [20]. Water loss in double mutants was examined either indirectly as leaf temperature differences visualized using infrared thermal imaging of detached leaves, or by rapid dehydration of rosettes (Figures 2 and 3).

The *aba3-1* and *ost1/snrk2e* single mutants displayed lower leaf temperatures than wild type due to their increased water loss upon dehydration, as previously reported [17], [20], [21] (Figures 2B and 3B). For the *aba3-1* mutant, this was due to its impaired ability to synthesize ABA, whereas the *ost1* mutant is affected in the ABA signaling pathway that induces stomata closure, downstream of ABA production. In contrast, *esk1* leaves were hotter and water loss was reduced compared to wild type, in accordance with the reduced evapo-transpiration reported by Bouchabke-Coussa and co-workers [14] (Figures 2B, C and 3B, C). Both *esk1-5 ost1* and *esk1-5 aba3-1* had leaf temperatures that were intermediate of those for the parental genotypes, as expected if the mutations affected independent pathways (Figures 2B and 3B). Nonetheless, intermediate leaf temperatures could also be obtained if the two genes act in partially overlapping pathways that modulate stomata opening. Similar results were obtained in water loss measurements on rapid dehydration of detached rosettes (Figures 2C and 3C). The *esk1* mutant plants lost only 30% of their weight as water, whereas *ost1* and *aba3-1* lost over 70%. Water loss from the *esk1-5 ost1*, *esk1-5 aba3-1* and *esk1-4 aba3-1* double mutants was always between these extremes.

As shown in Figure 2A, *esk1-5 ost1* and *esk1-5* mutant plants were the same size. As the *ost1* mutation does not affect plant stature, despite having open stomata, this indicates that the factor influencing plant size is not the degree of stomata opening. In contrast, both *esk1* and *aba3-1* mutants display dwarf phenotypes and the double mutant plants were even smaller (Figure 3A), indicating that the growth limitations caused by these mutations are additive. Thus, *ABA3* and *ESK1* are independent in the determination of plant size and act in different pathways. The small size of *esk1* is not, therefore, directly due to the high levels of ABA accumulation. Taken together the double mutant phenotypes demonstrate that ESK1 does not intervene in growth regulation and water loss through ABA and the high ABA levels in mutants is an indirect consequence of *esk1* mutation.

### Hydraulic conductivity is reduced from the *esk1* root apparatus

**Figure 4:**
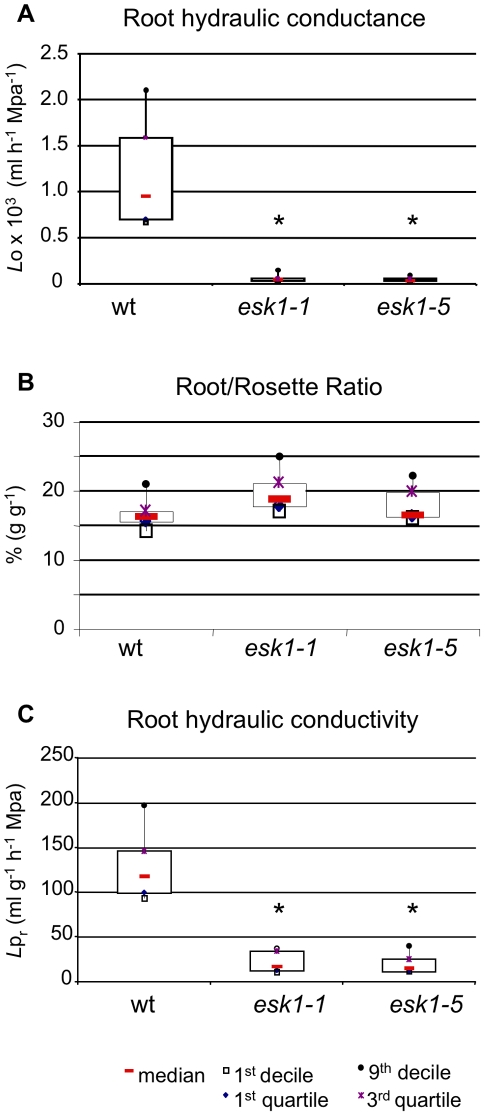
Hydraulic parameters of the root apparatus are modified in *esk1.* A. Root hydraulic conductance *L*o is expressed as ml of sap exudate per hour and per unit of exerted pressure. B. Root/rosette dry weight ratio. C. Root hydraulic conductivity is expressed as *L*o per unit of root mass. Wild type (wt). Black * indicates significant difference with wt (*p*-value<0.05).

The major driving force of plant water uptake is transpiration, which is controlled by stomata aperture. Entry of water into the plant is also regulated by the hydraulic conductance of the root apparatus and can potentially limit water availability in the shoots [22]. Given the reduced water loss, high leaf temperature and low RWC of *esk1* mutants [14] (Figures 2 and 3), we hypothesized that the overall water flux supplied by the xylem sap is reduced in *esk1* plants. In order to test this hypothesis, *esk1* root hydraulic conductance was measured and compared to wild type (Figure 4).

The root hydraulic conductance, or *L*o, represents the overall capacity of the root apparatus to transport water from the soil to the shoot. The root hydraulic conductivity, or *L*p_r_, is the conductance of a root surface unit: *L*o =  *L*p_r_*S, with S related to the surface of exchange between the root and its substrate. *L*o and *L*p_r_ have been measured for wild type and two *esk1* mutants, as well as the root/rosette dry weight ratio (Figure 4). Despite the small size of the *esk1* lines, the dry matter allocation between the root apparatus and the rosette was similar to that of wild type. The *L*o of *esk1* lines was exceptionally low: 6% and 5% of wild type for *esk1-1* and *esk1-5* respectively. Part of this low root conductance can be explained by the small biomass of the root apparatus. Nonetheless, the *L*p_r_ values for the *esk1* lines were also extremely low, indicating that the mutant root system produced a higher resistance to water transport from the soil to the hypocotyl. Thus, water transport is impaired in *esk1* mutants in comparison to wild type and probably induces the low evapo-transpiration and the reduced RWC observed in the mutants.

### The *ESK1* promoter specifically drives expression in vascular tissue

To determine the site of *ESK1* gene function, expression analyses were carried out using a 1 kb *ESK1* promoter region fused to the *GUS* reporter gene.

**Figure 5:**
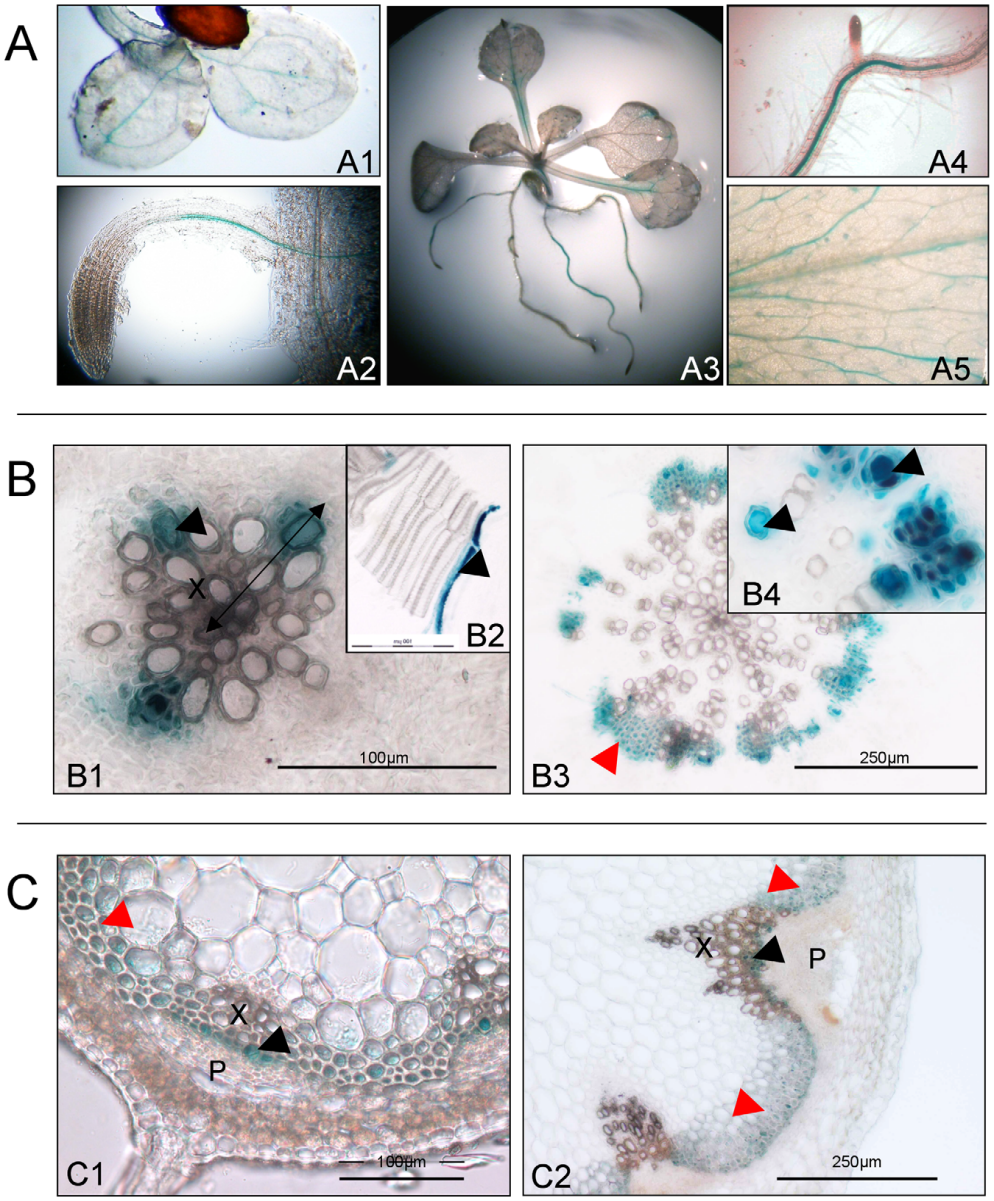
Histochemical analysis of *GUS* reporter gene expression from the *ESK1* promoter. A. Expression in whole plantlets 4 days after germination (A1, A2) and 10 days after germination (A3, A4, A5) with magnification of root (A4) and leaf (A5). B. Transverse section of hypocotyls, before (B1, B2) and after floral transition (B3, B4). C. Transverse section of stem (C1, C2). Double-headed arrow indicates the position of cross-section in B2. Red arrowheads indicate interfascicular fibers (B3) or sclerenchyma (C1, C2) and black arrowheads indicate newly-formed xylem cell (B1, B2, B4, C1, C2). X, xylem; P, phloem.

Seven independent insertion lines were analyzed and similar results for seedlings and mature plants were obtained: in all cases, expression was restricted to the vascular tissue and was initially observed a few days after germination, just after the appearance of the first differentiated vascular tissues, in the radicle and cotyledons (Figure 5A1, A3). In roots, GUS staining was absent from the meristematic and elongation zone and was only apparent in the differentiation zone (Figure 5A2). At ten days after germination, expression profile is still restrained to vascular tissues of all organs (Figure 5A3–A5).

More detailed characterization of *ESK1-*promoter driven expression within the vascular tissue was performed on three independent homozygous transgenic lines, using sections of hypocotyls and stems. As shown in Figure 5 (B1, B2, C1), GUS staining was restricted to the cells undergoing xylem vessel differentiation, in both hypocotyls and stems. Furthermore, *ESK1* also appeared to be expressed in the interfascicular parenchyma in the stem (Figure 5C1, C2). After floral induction, GUS staining was also observed in the fibers of the hypocotyls as well as xylem vessels (Figure 5B3, B4).

These results reveal that the *ESK1* gene is specifically expressed in tissues undergoing secondary cell wall deposition.

### *esk1* mutants show altered vascular apparatus morphology

As *ESK1* expression was specific to vascular tissue and *esk1* mutants were defective in water transport, we conducted a detailed analysis of vascular tissue structure in different *esk1* mutants.

**Figure 6:**
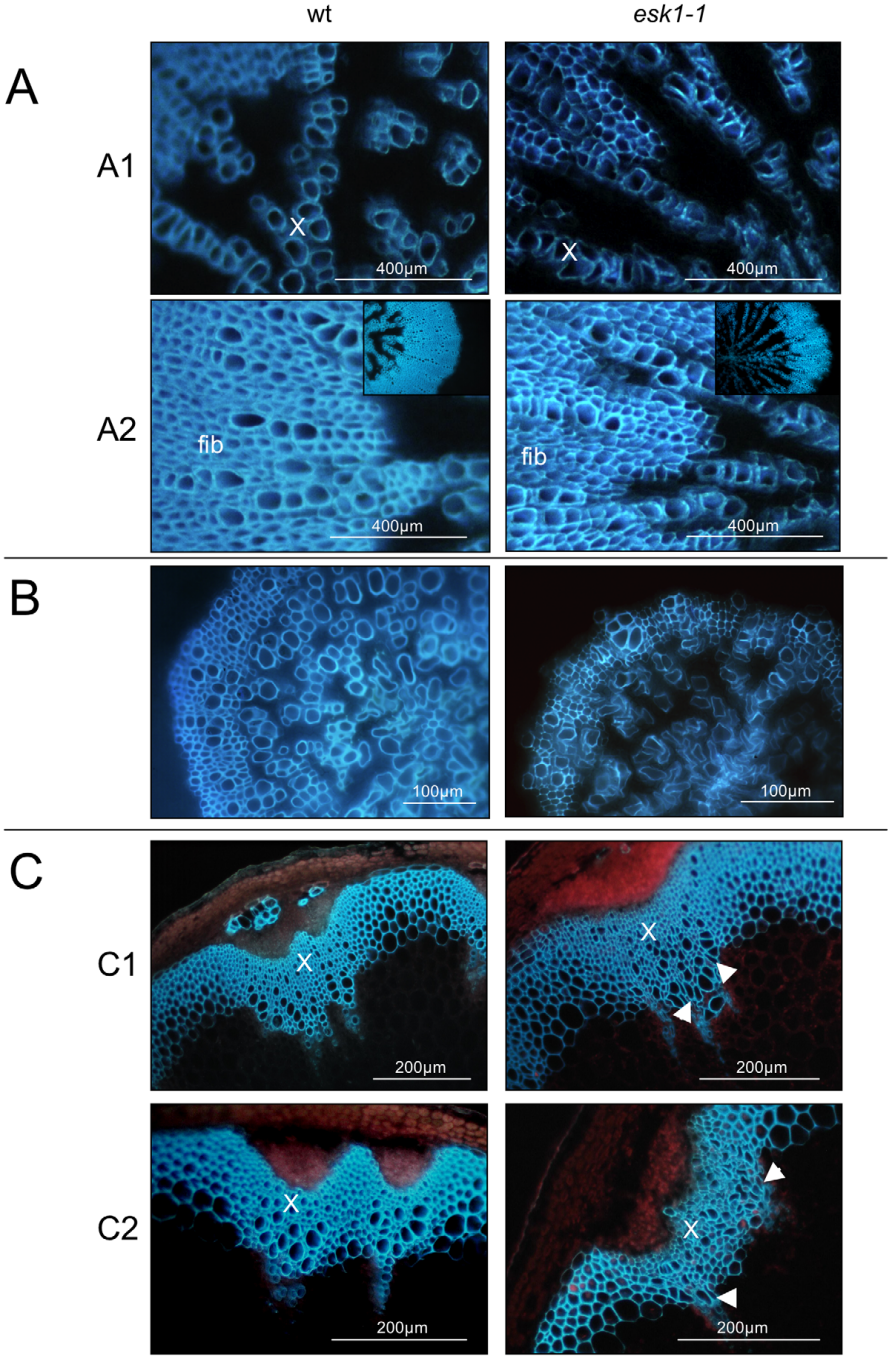
Comparison of vascular tissue of *esk1-1* and wild type, in hypocotyls, roots and stems. Vascular tissue is shown for *esk1-1* on the left hand side and for wild type (wt) on the right hand side. A. Transverse section of hypocotyl observed under UV light. A1, fibers (fib); A2, xylem cells (X). B. Transverse section of root observed under UV light. C. Transverse section of the stem base (C1) and at mid-height (C2) observed under UV light. Arrowheads indicate collapsed xylem.

Similar results were obtained with the three mutants studied and results obtained with *esk1-1* are presented. Transverse sections were performed on hypocotyls, roots and stems of 6 week-old plants grown on soil without water deficit (Figure 6). Xylem in vascular bundles of *esk1* roots and hypocotyls appeared collapsed (Figure 6A1, B). In hypocotyls, fibers also seem to be affected: cells appeared angular and the ring of fibers that surrounded xylem vessels seemed less regular than in wild type (Figure 6A2). This phenotype was reminiscent of the *irx* mutants that have collapsed xylem [23]. When stems were only a few centimeters high, their xylem vessels seemed unaffected (Figure S1). However, at later stages, when they developed lateral branches, irregularly shaped cells and collapsed xylem elements were observed in stems at both the base (Figure 6C1) and mid-height level (Figure 6C2).

In view of this phenotype, we hypothesized that the very low *esk1* root hydraulic conductivity was due to restricted water transport through collapsed vessels. This is defined by Poiseuille's law, in which the major factor affecting the hydraulic conductance of a tube is its diameter. The theoretical conductivity of root tracheary elements (TE) was calculated for *esk1* mutant and wild type, based on maximal diameter measurements obtained from transverse sections of root. As shown in Table 1, the flux calculated for *esk1* mutants is approximately five-fold lower than that of wild type. This establishes a direct consequence of the *irx* phenotype observed for *esk1* mutants on hydraulic conductivity through the root apparatus.

**Table 1:** Low *esk1* root hydraulic conductivity is mainly explained by a reduction in tracheary element diameter.

	wt (n = 19)	*esk1-1* (n = 18)	*esk1-5* (n = 19)
Mean radius	8.14	5.08	5.93
Standard error	0.14	0.27	0.16
Max radius	10.16	7.00	6.81
Max conductivity	4.18	0.94	0.84
% of wt	100	22.6	20.2

The 4 to 5 larger xylem vessels of 6 week-old plants of *esk1* mutant alleles and wild type (wt) sections were analyzed for the maximal circular radius. The maximal conductivity (g) of the vessels was calculated according to Poiseuille's law.

R = (8.η)/(π.r^4^) where g = 1/R (m^2^/s/MPa), η is the water viscosity coefficient (1E^−9^ MPa/s) and r is the maximal radius (µm).

Max conductivity: maximal hydraulic conductivity calculated.

Mean radius: mean of all radius measured (µm).

Max radius: maximal radius measured (µm).

### The chemical composition of *esk1* xylem is different from wild type

**Figure 7:**
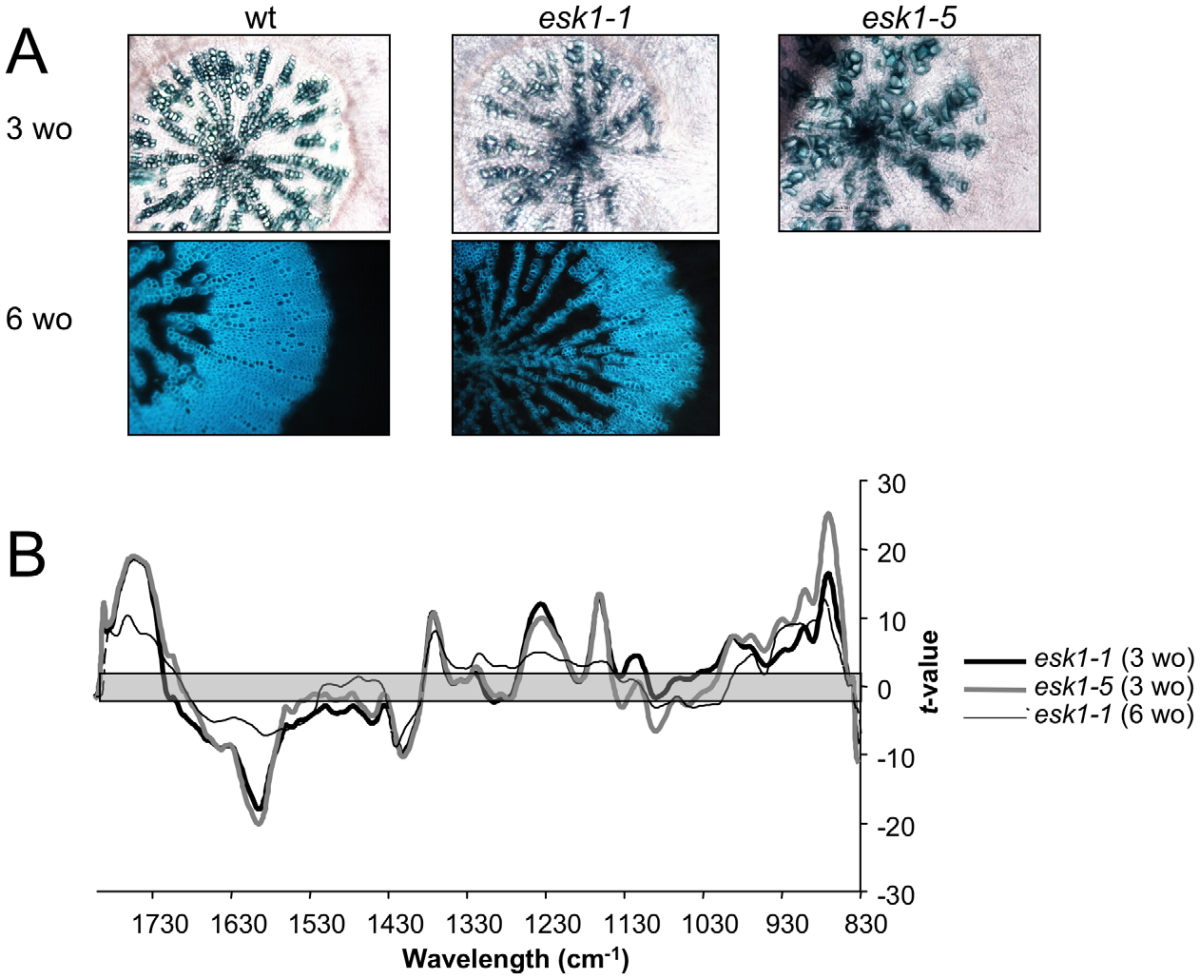
The structure and composition of *esk1* xylem is modified in hypocotyls. A. Transverse section of hypocotyls from 3 week-old (3 wo) and 6 week-old (6 wo) *esk1-1* and *esk1-5* plants, with lignin stained in green with Carmine-green (3 wo), or lignin fluorescing under UV light (6 wo). B. Comparison of FTIR spectra obtained from xylem in hypocotyl sections of 3 week-old (3 wo) or 6 week-old (6 wo) wild type and *esk1* plants. A Student's *t*-test was performed on absorbance values of wild type versus mutant and plotted against wave numbers. The grey zone, between -2 and +2, corresponds to non-significant differences (*p*-value<0.05) between the two genotypes tested. Significant positive *t*-values indicated a higher absorbance value in wild type than in *esk1* mutants.

**Figure 8:**
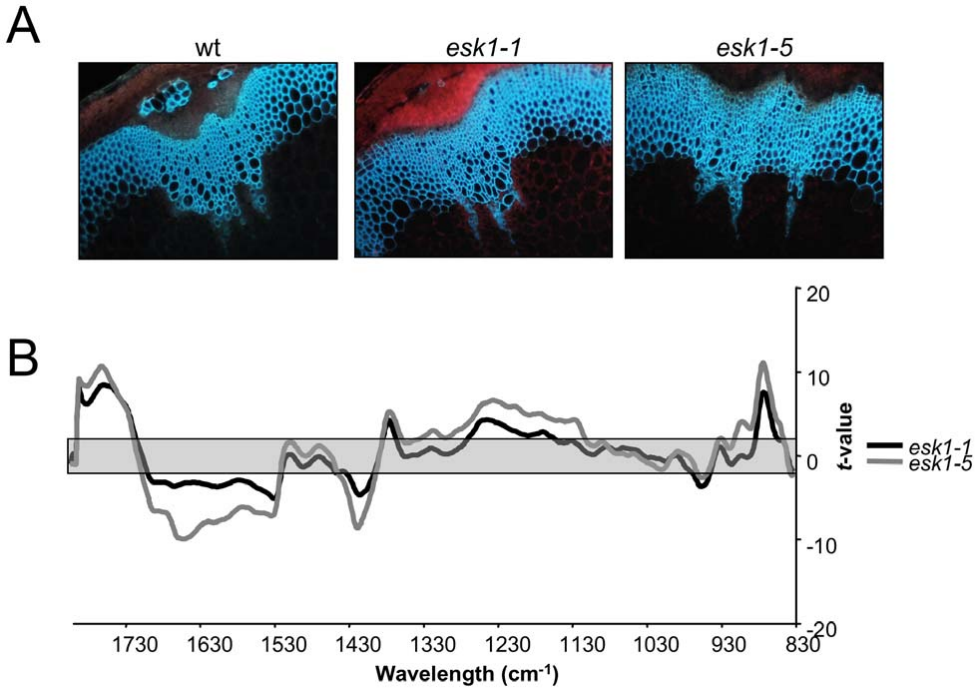
The structure and composition of *esk1* xylem is modified in stems. A. Transverse section of stems from *esk1-1* and *esk1-5* plants, with lignin fluorescing under UV light. B. Comparison of FTIR spectra obtained from xylem in basal stem sections of wild type and *esk1* plants. A Student's *t*-test was performed on absorbance values of wild type versus mutant and plotted against wave numbers. The grey zone, between −2 and +2, corresponds to non-significant differences (*p*-value<0.05) between the two genotypes tested. Significant positive *t*-values indicated a higher absorbance value in wild type than in *esk1* mutants.

The chemical composition of the vascular tissue of hypocotyls at two developmental stages and the stem base was examined using FTIR spectral analysis performed on the xylem of transverse sections. Two representative *esk1* null mutants were analyzed and a Student's *t*-test was performed, corresponding to the differences between wild type and *esk1* spectra. For graphical representation, *t*-values were plotted against wave numbers to determine the significance of the differences at each wavelength (Figure 7B and 8B).

In accordance with the severe vascular tissue defects observed in both hypocotyls (Figure 7A) and stems (Figure 8A), the infrared absorbance spectra from *esk1* mutants were different from those of wild type. Furthermore, *esk1-1* FTIR profiles obtained from stems (Figure 8B) and from hypocotyls before and after fiber appearance (Figures 7B) presented the same differences to wild type indicating that the composition of the xylem cell walls was modified in a similar manner in these different tissues.

**Figure 9:**
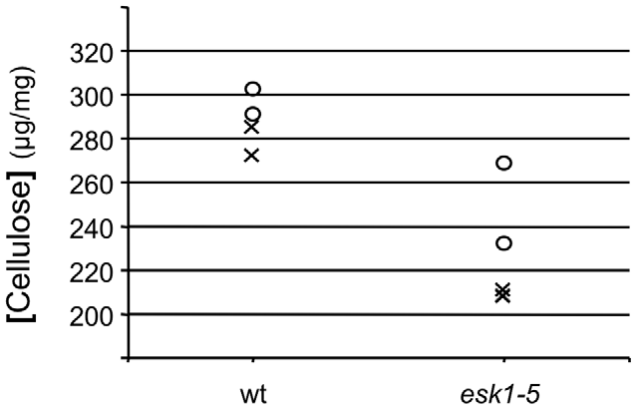
Crystalline cellulose content is reduced in *esk1-5* hypocotyl. Cellulose contents expressed as % of hypocotyl dry weight. Data from 4 measurements are plotted. The decrease in *esk1-5* cellulose content compared to wild type (wt) was significant using Mann and Whithney non parametric test (*p*-value<0.05).

Significant differences were observed in the absorption at wavelengths from 1800 cm^−1^ to 1700 cm^−1^, which correspond to ester linkages [24] or esterified pectins [25]: *esk1* absorbance was reduced compare to wild type. In addition, a highly significant peak was observed at wavenumbers around 1240 cm^−1^, assigned to C-O vibrations in pectic polysaccharides [25]. Other major differences were in a series of wavelengths between 1000 cm^−1^ and 830 cm^−1^, associated with crystalline polysaccharide components such as cellulose, and suggest a reduction in the amount of crystalline cellulose [24]. For example, 898 cm^−1^ corresponds to the β-linked glucan polymers [25]. A direct measurement of crystalline cellulose carried out on 6 week-old hypocotyls and stems confirmed that the *esk1* mutant contains lower levels of cellulose than the wild type (Figure 9 and data not shown). In addition, significant differences were noted between wild type and *esk1* mutants at the wavelengths around 1370 cm^−1^ that are associated with CH_2_ stretches of cellulose and 1157 cm^−1^, attributed to cellulose C-O-C linkages [25]. Finally, significant differences in absorbance at other non-assigned wavelengths were also observed between wild type and mutants.

Interestingly, in the transcriptome data previously obtained for *esk1-5* [14], no “core genes” participating directly in cellulose, hemicellulose, pectin or lignin biosynthesis or modification were down-regulated in *esk1-5* background. However, At1g23205, annotated as a pectin methyl esterase inhibitor (PMEI) was expressed 6.6 times less in *esk1-5* than in wild type, in accordance with the reduction in esterified pectins indicated by FTIR analysis.

## Discussion

*esk1* mutants were initially described as freezing tolerant without cold acclimation [12] and ESK1 was proposed to be a negative regulator of freezing tolerance [26]. Later studies also revealed altered responses to other stresses such as drought and salt, thereby revoking the specificity of its action. Instead, ESK1 was proposed to play a role in water uptake or circulation [14], [15]. Nevertheless the precise function of ESK1 in these processes remained to be determined.

### ESK1 plays a role in xylem layout

Xylem conducts water and nutrients from the soil to sink organs. Its differentiation occurs in two steps which seem to be coupled [27]. During the first step, only xylem vessels are generated, and in the second, corresponding to secondary growth, xylem and fibers are formed. TE then undergo programmed cell death (PCD), leading to functional cell corpses. These empty cells are connected end-to-end by perforated cell plates that allow fluid transport and can support the turgor pressure generated by transpiration without collapsing [28]. Thus, differentiation of TE involves several steps: cell expansion, followed by the deposition of a thick secondary cell wall and cell death [29].

In seedlings grown *in vitro*, *GUS* expression from the *ESK1* promoter was observed in vascular tissue a few days after germination and may coincide with the occurrence of secondary cell wall deposition, even though the exact time point of the onset of secondary vascular development has not been determined in Arabidopsis [30]. A close inspection of *ESK1*-promoter driven staining pattern in the xylem of hypocotyl before floral induction revealed that (i) only the last cell of the xylem cell file was stained and (ii) stained cells presented morphological differences, from dense stained cells, to cell corpses possibly corresponding to different stages of xylem vessel differentiation, rather than PCD. Moreover, at later stages of development, staining was also apparent in interfascicular fibers suggesting that *ESK1* expression occurs in tissues undergoing or that will undergo secondary cell wall deposition and not specifically in TE cell lines (Figure 5).

Cytological analysis revealed that *esk1* mutants presented a strong *irx* phenotype in roots, hypocotyls and stems (Figure 6). The localization of *ESK1*-promoter driven expression in TE and fibers is in agreement with the collapsed xylem vessels and disorganized fibers observed for *esk1* mutants. Several *irx* mutants have been shown to be affected in their secondary cell wall synthesis and some of them display reduced resistance to compressive force [23]. For instance, *irx1/cesa8*, *irx3/cesa7* and *irx5/cesa4* mutants are affected in subunits of the cellulose synthase complex [31], [32], [33], [34]; *irx8* is deficient in hemicellulose [35]; *cad-c cad-d* double mutant is affected in cinnamyl alcohol dehydrogenase and has 40% less lignin than wild type plants as well as collapsed xylem elements [36]. These *irx* mutants, apart from *cad-c cad-d*, also have a reduced stature. Moreover *irx3/cesa7* and *cad-c cad-d* have reduced rigidity, indicating the importance of the composition of the plant cell wall in determining the physical properties of the plants.

In addition, the expression patterns observed for *ESK1* were similar to those for reporter genes under the control of transcription factor promoters that are involved in secondary cell wall synthesis [37]. Therefore, ESK1 may participate in the process leading to secondary cell wall deposition.

### The *esk1* rosette suffers from a defect in water supply from the root apparatus

As xylem vessels are conducting tubes that transport water, defects in their shape may have consequences on water distribution. Water supply is determined by the transport capacity of the root system, or root hydraulic conductance, with the degree of stomata opening controlling the rate of the transpiration driven water uptake into plants. The *esk1-5* mutant had high levels of ABA, causing stomata closure and consequently low levels of evapo-transpiration (Figures 1, 2 and 3). In the *esk1-5 ost1* and *esk1-5 aba3-1* double mutants, water loss and leaf temperature, were intermediate between those of the single mutants (Figure 2B, C and 3B, C). This higher evapo-transpiration compared to *esk1* single mutants was likely due to increased opening of stomata, which are, therefore, functional in *esk1.* Thus, the reduced levels of evapo-transpiration observed in *esk1* mutants were not due to permanently closed stomata. We hypothesized that this could be because water uptake or transport to the vegetative tissues is reduced by the *esk1* mutation.

When hydraulic conductivity was assessed, this was found to be lower for *esk1* mutant roots (Figure 4 and Table 1). Water uptake by the roots occurs through radial transfer from the soil to the xylem vessels through the apoplast, the symplast or through the cell-to-cell pathway where it crosses cell membranes through water channels named aquaporins [38], [39]. Apoplastic, symplastic or aquaporin water transport modification could partly explain the lower *L*pr measured for *esk1* roots compared to wild type (Figure 4 and Table 1). Nonetheless, transcriptome analyses did not find significant alterations in aquaporin transcript abundance in *esk1-5* plants [14]. A reduction in root system biomass could also cause a moderate decrease in water flow (Figure 4A, C), as would modifications to the root structure that generate resistance to water transport from the soil to aerial parts.

Once water has reached the xylem, its flux largely depends on the diameter of the vessels. The fact that the shape of the xylem vessels in *esk1* roots was strongly altered could cause a higher resistance of *esk1* vessels to water flow, independent of the water supply status, in agreement with the particularly low hydraulic conductivity measured for the *esk1* lines (Figure 4C). The maximal conductivity calculated using Poiseuille's law predicts a decrease of 80% in *esk1* compared to wild type due to decrease in the diameter of roots vessels (Table 1). This reduction is of the same order of magnitude as the decrease observed in root hydraulic conductivity measured. This suggests that the diameter of the xylem vessels is the main factor limiting water flux in xylem in *esk1* mutants. By contrast, in maize the radial resistance is usually rate-limiting and not the longitudinal hydraulic resistance within the xylem [39]. These results suggest that the origin of the disrupted water uptake and water circulation through *esk1* xylem would be the alterations observed in the vessels' structure. Therefore, ESK1 appears to be necessary for the correct formation of functional xylem vessels.

### The *esk1* stressed phenotype is likely due to its TE default

Our results tend to show that, as a result of collapsed TE, the overall volume of water circulating in *esk1* xylem is greatly reduced.

In previous studies, the *esk1-5* mutant was described as constitutively stressed with regard to its physiology: smaller stature of the plants, reduced RWC and transpiration rate, better WUE, similar transcriptomic profile and metabolomic data under control and drought conditions [14], [15]. The increased osmolyte concentrations observed in *esk1* lines compared to wild type [14], [15] would explain the freezing-tolerant phenotype described by Xin and co-workers [26]. In the current study, we showed that ABA levels in *esk1* mutants were higher than those of wild type in non-stressed conditions, which would be incoherent with a role for ESK1in stomata closure through ABA signaling downstream [40]. Analysis of the double mutant *esk1-5 aba3-1* showed that this was not due to a direct role of ESK1 in ABA biosynthesis. Indeed, the double mutant phenotypes demonstrated that ESK1 did not intervene in growth regulation and water loss through ABA and that high ABA levels were an indirect consequence of *esk1* mutation. Moreover, double mutant studies and root *L*p_r_ measurement confirmed that *ESK1* could contribute to water circulation in the plant, as suggested earlier [14], [15]. This conclusion is consolidated by the fact that the size of *esk1-5 ost1* double mutant is identical to *esk1-5* (Figure 2A) and is, therefore, independent of the level of evapo-transpiration by the plant and only dependant on the amount of water in the plant. In addition, *esk1-5 aba3-1* double mutants were smaller than both dwarf single mutants due to the combined effects of ABA deficiency and reduced water transport.

Like *esk1*, *irx1*/*cesa8* has a reduced stature and high ABA levels, together with collapsed xylem and reduced water loss on detached leaves [32]. Chen and coworkers also observed an enhanced drought and osmotic stress tolerance in *irx1*/*cesa8* mutant compared to wild type [32]. Moreover, *irx14* mutant, affected in a glycosyl transferase involved in cell wall glucuroxylan biosynthesis, has recently been shown to exhibit drought tolerance [41]. It will be interesting to see whether this is a general phenomenon among *irx* mutants.

By integrating all the aspects of the *esk1* phenotype, a model can be obtained were the low hydraulic conductance of the root apparatus, due to xylem vessels malformation, induces an hydraulic signal to the shoot, resulting in ABA synthesis [42]. The consequences of high ABA levels are well documented, and include stomata closure, low evapo-transpiration and synthesis of metabolites like soluble sugars and proline.

### Xylem malformation in *esk1* is accompanied by alterations in secondary cell wall composition

*irx* phenotypes have been reported for mutants affected in their cell wall composition. FTIR analyses were performed on *esk1* sections targeting xylem, which are mainly composed of secondary cell wall. We observed differences between wild type and *esk1* that were attributed to reduced pectin esterification and crystalline cellulose in the mutant. The later was confirmed by crystalline cellulose measurements (Figures 7, 8 and 9 and data not shown).

Although pectins are mainly present in primary cell walls [43], [44], pectin methylesterification appears to be a prerequisite for lignin modifications during secondary cell wall deposition in xylem cells [45]. A feedback loop which would interconnect the control of cellulose and pectin biosynthesis has already been suggested to participate in the homeostasis of wall rigidity [46]. Compensation mechanisms have also been observed between primary and secondary cell walls, when studying the *irx3* mutant affected in a secondary cell wall specific cellulose synthase gene [31]. It is also worth noting that comparative FTIR spectroscopy performed on the *irx1* mutant clearly demonstrated that the cell wall modifications encountered in *esk1* mutants are not those of a cellulose synthase deficient mutant (Figure S2).

All the modifications observed in *esk1* mutants would therefore be consistent with a role for ESK1 in the deposition or maintenance of a functional secondary cell wall, specifically in xylem and interfascicular fibers, its defect inducing a strong *irx* phenotype. Indeed, the altered secondary cell wall composition may result in a modification of the mechanical properties of the TE, so that resulting cell walls cannot provide enough strength to endure negative pressure, therefore inducing an *irx* phenotype [23].

Recently, sequence analysis predicted ESK1 to be part of a novel group of proteins named the PC(Pmr5-Cas-1p)-esterase family. Members of this family share a putative N-terminal acylesterase domain and ESK1 was proposed to modify cell wall glycans through carbohydrate acylation [47]. ESK1 also contains a C-terminal Domain of Unknown Function 231 (DUF231) [26]. Bischoff and co-workers found that members of the DUF231 gene family like *POWDERY MILDEW RESISTANCE 5* (*PMR5*), *TRICHOME BIREFRINGENCE* (*TBR*) *TBR-LIKE 3* (*TBL3*) and *ESK1*, harbor another plant-specific domain (TBL domain) that contains a conserved GDSL motif common to certain esterases and lipases [48]. Interestingly, FTIR analysis and PME activity measured for *tbr* and *tbl3* etiolated seedlings also indicated reduced levels of esterified pectins in their cell walls compared to the wild type [48]. Moreover, secondary cell wall cellulose contents were reduced in *tbr* and *tbl3.* Bischoff and co-workers concluded that the TBL/DUF231 genes might be pectin-binding proteins that are involved in the overall cell wall chemical balance [49]. Bischoff and Scheible also propose that ESK1 should be renamed TBL29 (personal communication).

Nevertheless, we cannot exclude a more general action of ESK1 in xylem formation, as such broad impact on secondary cell wall chemical composition has not previously been reported for any cell wall mutant. The precise function of proteins harboring a DUF231 remains to be determined.

### Conclusion

The data presented here demonstrate that *ESK1* plays a major role in the formation of functional xylem vessels, which has dramatic consequences on water transport. *ESK1* is expressed in cells that develop into xylem and TE are collapsed in *esk1* mutants, a characteristic feature of *irx* phenotype. We establish for the first time in this study a clear link between this TE phenotype and the low water conductance, which explains their small stature, low evapo-transpiration, the stressed plant state and the stress tolerance previously reported [12], [14], [15]. Our results favor the hypothesis that ESK1 is a protein involved in cell wall deposition, maturation or regulation, as suggested for other members of the DUF231 family [47], [48], [49], [50]. It is also possible that the defect in ESK1 could influence earlier steps with consequences on the correct assembly of cell walls in xylem and interfascicular fibers.

Identification of the molecular function of ESK1, its involvement in stress responses and its potential role in cell wall formation will require further investigation.

## Materials and Methods

### Plant material and growth conditions

*esk1-1* was isolated by Xin *et al.* [26] and *esk1-4* and *esk1-5* have been described by Bouchabke-Coussa *et al.* [14]. *ost1*/*snrk2e* [51] was kindly provided by Dr J. Leung, *aba3-1* was identified by Leon-Kloosterziel *et al.* [17] and *irx1-1* was described by Turner *et al*. [23]. *esk1-1*, *esk1-4*, *esk1-5*, *ost1*/*srk2e* and *aba3-1* are in the Col-0 genetic background, *irx1-1* is in the L*er* background.

### Abiotic stress

For ABA measurements, control culture conditions and mild drought stress were applied to individual plants, grown on Fertiss® propagation plugs. Seeds were stratified 3 or 4 days at 4°C in 0.1% (w/v) agar and then two seeds were sown onto each propagation plug. After the emergence of cotyledons, seedlings were removed to leave one plant on each plug. Plants were grown in controlled conditions in a growth chamber (22°C, light intensity 180 µmol m^−2^ s^−1^, 16 h photoperiod, 70% RH). Stress was applied at bolting: twenty-five days after sowing in our conditions. Each propagation plug was adjusted daily to the target substrate saturation with nutritive solution: 60% (w/v) for the control and 30% (w/v) for mild drought stress as previously described Bouchabke-Coussa *et al.* [14]. Plants were harvested after 7 days of stress implementation.

### Infrared thermography and water-loss assays

Rapid dehydration assays were carried out using 3-week-old plants grown on propagation plugs in a growth-chamber (22°C, light intensity 180 µmol m^−2^ s^−1^, 16 h photoperiod, 70% RH). Four rosettes per genotype were cut from their root system and water loss was measured as described previously [52].

For leaf temperature measurements, plants were grown for 4 weeks in soil in a glasshouse (22°C, minimum 13 h photoperiod, maximum light intensity of 500 µmol m^−2^ s^−1^). In a growth chamber (25°C, 150 µmol m^−2^ s^−1^, 50% RH) leaves from well-watered plants were detached, placed abaxial-side uppermost and images acquired using an A320 infrared camera equipped with a 45° lens (FLIR Systems; http://www.flir.com).

### ABA content determination

Four independent experiments were carried out using plants at inflorescence emergence, either subjected to mild water deficit [14] or controls. Triplicate measurements were carried out for each condition and each measurement was performed using tissue from a pool of three rosettes. Tissue was freeze-dried prior to extraction of ABA.

Samples were weighed and 33 ng of a standard ABA-d_4_ (Euriso-Top SA, France) were added as an internal standard. Freeze-dried material was ground in 3 ml acetone/water/acetic acid (80/19/1; v/v/v), then centrifuged (4630 *g*, 4°C, 3 min). After re-extraction of the pellets with 1 ml of extraction solvent and sonication for 20 min (25 Hz), the supernatants were combined and concentrated under nitrogen. The dry extract was dissolved in 150 µl acetonitrile/water (50/50; v/v), filtered and analysed by HPLC–electrospray-tandom mass spectrometry (HPLC-ES-MSMS). Sample components were separated on a reverse-phase column (Uptisphere C18 5 µm, 150×2 mm i.d, Interchrom) using a Waters 2695 separation module (Alliance) (Waters, Milford, MA, USA) equipped with a Waters 2487 dual UV detector, with a flow-rate of 0.15 ml/min and a binary gradient: acetonitrile 0.5% acetic acid (v/v) (A) and acetic acid 0.5% (v/v) (B). Typically, the solvent gradient was programmed as following: 0–5 min 20% A, 5–15 min 65% A, 15–20 min 100% A, before returning to the initial composition at 30 min. Separated molecules were ionized in the ESI source and analyzed with a Waters Quattro LC triple quadrupole mass spectrometer (Waters, Milford, MA, USA) operating in a Multiple Reaction Monitoring (MRM) scanning mode. Instrument parameters were set as follows: capillary 2.75 kV (negative mode), extractor 2 V, source block and desolvation gas temperatures 120 and 350°C, respectively. Nitrogen was used to assist the nebulisation and the desolvation (250 and 450 L/h respectively), argon was used as collision gas at 3.5 10^−3^ mbar. The parameters used for MRM quantification of ABA-d_4_ and ABA in positive mode were: cone potential 16 V, collision energy 10 eV for two transitions used m/z 267>251 and 263>247 respectively. The limit of detection (LOD) and limit of quantification (LOQ) were calculated from calibration curve and sample using the Quantify module of MassLynx version 4.1 software. Typically, for a 5 µl injection of sample prepared with 33 ng internal standard and reconstituted in 150 µl of 50/50 acetonitrile/H_2_O (v/v), the respective LOD and LOQ are 1 and 3 pg/mg dry mass.

Data from the four independent experiments were combined for the statistical analysis. Multiple-samples comparisons were performed between genotypes for each treatment independently. Analysis of the effect of two variables, i.e. genotype and water treatment, and their interactions on the ABA concentration was performed by 2-D ANOVA for each pairs of genotypes. ANOVAs and multiple-samples comparisons were performed with the Statgraphics® software. Multiple-samples comparisons were carried out using the Fisher test (Least Significant Difference) at the 5% threshold. Box-plots graphs were performed with tools created by the Anastats association, available free at http://www.viesanimales.org/stats/Download.htm.

### Reporter constructs, GUS assays and cytological observations

An approximately 1 kb fragment of the *ESK1* promoter was amplified from wild type Col-0 genomic DNA using 5′ attB1-TGGTTGGTGCCGTATACATA 3′ and 5′ attB2-CCAAGGTTGCATCTGTTTGT 3′ primers, where attB1 and attB2 contain sequences complementary to the Gateway™ recombination sequences. The PCR product was cloned in the pDONR207™ gateway entry vector (Invitrogen™) via BP recombination reaction. Subsequently an LR recombination reaction was carried out to introduce the fragment into the pBI101-R1R2 binary destination vector (F. Divol, J.-C. Palauqui and B. Dubreucq, Institute Jean-Pierre Bourgin, INRA, Versailles, France, unpublished data) as a fusion with the *GUS* reporter gene. Electro-competent C58C1 *Agrobacterium tumefaciens* were transformed with this construct, which was then used for agroinfiltration of wild type Arabidopsis flower buds. Seven independent transgenic lines, each with a single homozygous T-DNA insertion, were retained for subsequent analyses.

Histochemical detection of *GUS* expression was performed as described by Jefferson *et al.* [53]. Potassium ferricyanide/potassium ferrocyanide was used and concentration adjusted (from 1 to 3 mM) depending on the insertion line, the organ and the developmental stage considered. Samples were observed by light microscopy (LEICA DMR B DIC). For observation of hypocotyls or stems or GUS stained, *esk1* and wild type samples were embedded in 8% agarose and 70 µm sections cut using a vibratome. *esk1* and wild type sections were observed under UV light, where lignified tissues appears fluorescent, or under white light when stained with carmine-green which gives green coloration to lignin and pink coloration to cellulose [54].

### Hydraulic conductance

Root water transport measurements were performed essentially as described in Javot *et al.* [55]. Freshly excised root organs were inserted into a pressure chamber with the hypocotyls threaded through the lid of the chamber. Upon pressurization, the flow rate of the exuded sap was measured and plotted as a function of the pressure imposed. The gradient of the graph obtained provided the hydraulic conductance measure, or *L*o, which was then adjusted with respect to root dry weight in order to obtain the root hydraulic conductivity, or *L*p_r_. Data were obtained from 13 measurements for wild type, 9 measurements for *esk1-1* and 5 measurements for *esk1-5*.

Box-plots graphs were performed with tools created by the Anastats association, available free at http://www.viesanimales.org/stats/Download.htm.

For calculation of root maximal conductivity, ImageJ software was used to measure the maximal radius of the 4 to 5 larger xylem vessels. 4 images of root sections observed under UV light, from 3 different plants were used. The maximal conductivity (g) of the vessels was calculated according to Poiseuille's law.

R = (8.η)/(π.r^4^) where g = 1/R (m^2^/s/MPa), η is the water viscosity coefficient (1E^−9^ MPa/s) and r is the maximal radius (µm).

### FTIR spectroscopy

Analyses were carried out on xylem tissue from hypocotyls or the basal region of branched stems using 50 µm thick vibratome sections of agarose-embedded tissue. For each genotype, 5 to 6 sections from 3 different plants were analysed. FTIR spectra were collected from a 50 µm x 50 µm window targeting xylem vessels; normalization of the data and statistical analysis (Student's *t*-test) were performed as described in Mouille *et al.* [24].

### Crystalline cellulose extraction and dosage

Plant material was harvested and stored in absolute ethanol. Hypocotyl or stem pieces were ground and incubated twice in 70% ethanol for 60 min at 70°C. Pellets were then washed in acetone for 2 min at room temperature then vacuum-dried. After weighing samples, crystalline cellulose content was determined as described in Scott and Melvin [56] and Updegraff [57]. Two independent experiments were carried out with two replicates in each. A Mann and Whitney non parametric test was used to compare cellulose content between the two genotypes.

Mann and Whitney non parametric test and box-plots graphs were performed with tools created by the Anastats association, available free at http://www.viesanimales.org/stats/Download.htm.

## Supporting Information

Figure S1 **Vascular tissue structure in young stems of *esk1* and wild type.** Transverse section of young stems (2 to 3 centimeters high) from wild type (A, wt) and one representative *esk1* mutant plant (B, *esk1-1*), with lignin stained in green with Carmine-green.
(TIF)

Figure S2 **The structure and composition of *esk1-1* and *irx1-1* xylem are different.** Comparison of FTIR spectra obtained from xylem in basal stem sections of *irx1-1* and *esk1-1* plants and their respective wild types, L*er* and Col-0, respectively. A Student's *t*-test was performed on absorbance values of wild type versus mutant and plotted against wave numbers. The grey zone, between −2 and +2, corresponds to non-significant differences (*p*-value<0.05) between the two genotypes tested.
(TIF)

Table S1 **Statistical comparisons of ABA measurements from wild type (wt), *esk1* and *aba3-1* mutants.**
(XLS)
