# Supplementary figures and images for: ESKIMO1 Disruption in Arabidopsis Alters Vascular Tissue and Impairs Water Transport
Source: PLoS One. 2011 Feb 1;6(2):e16645. doi: 10.1371/journal.pone.0016645 (PMC3052256; doi:10.1371/journal.pone.0016645)

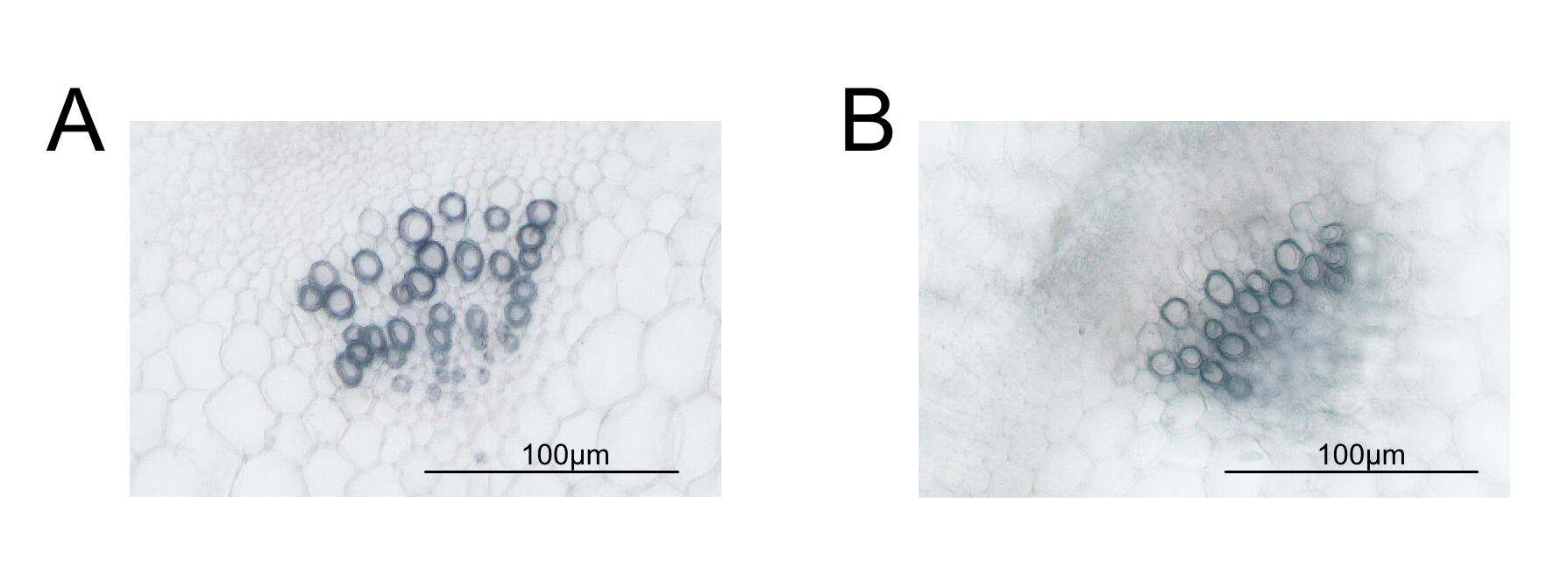

Supplement: Figure S1 — Vascular tissue structure in young stems of esk1 and wild type. Transverse section of young stems (2 to 3 centimeters high) from wild type (A, wt) and one representative esk1 mutant plant (B, esk1-1), with lignin stained in green with Carmine-green. (TIF) [file pone.0016645.s001.tif]

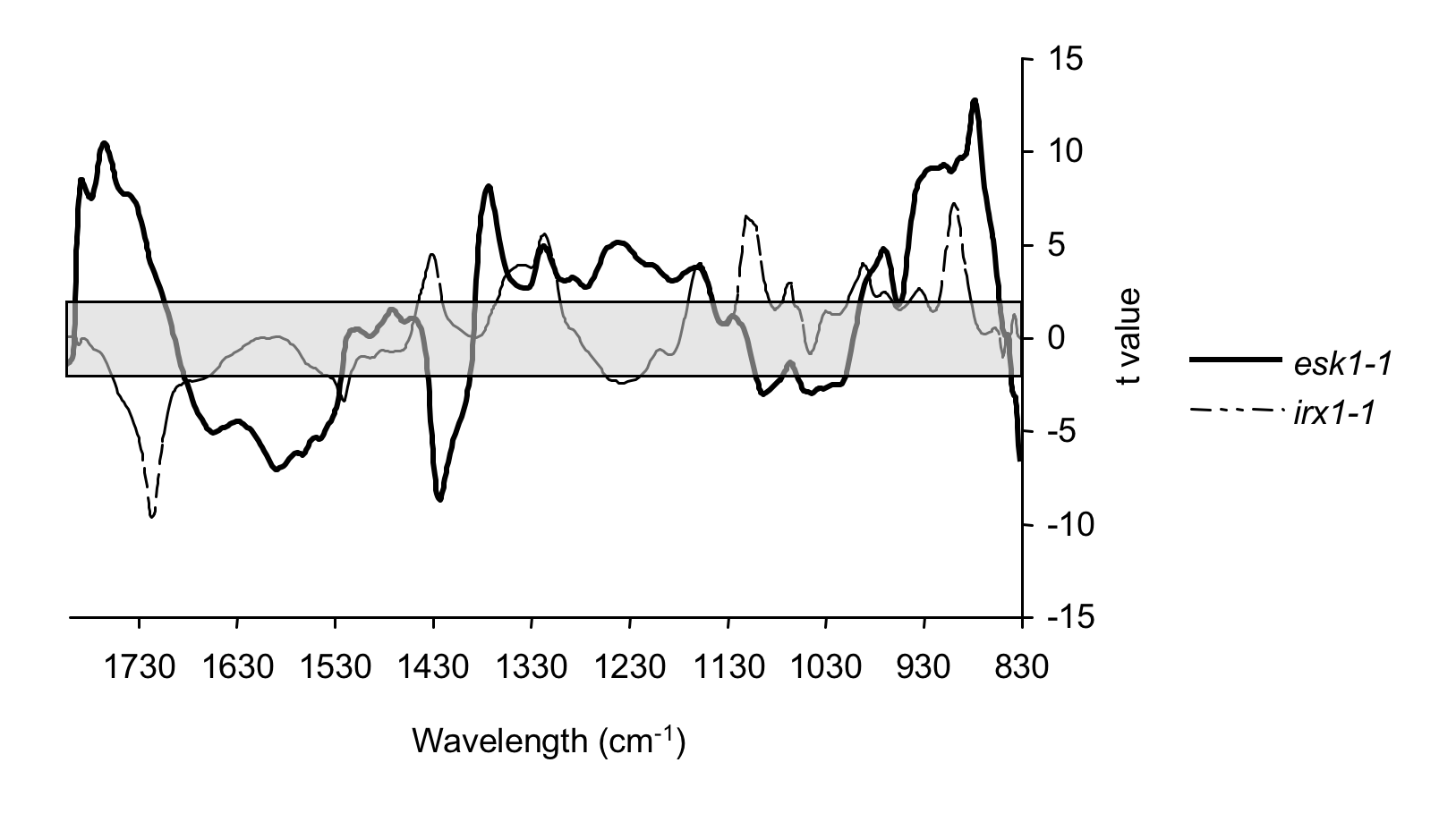

Supplement: Figure S2 — The structure and composition of esk1-1 and irx1-1 xylem are different. Comparison of FTIR spectra obtained from xylem in basal stem sections of irx1-1 and esk1-1 plants and their respective wild types, Ler and Col-0, respectively. A Student's t-test was performed on absorbance values of wild type versus mutant and plotted against wave numbers. The grey zone, between −2 and +2, corresponds to non-significant differences (p-value<0.05) between the two genotypes tested. (TIF) [file pone.0016645.s002.tif]
